# Migration-related changes in smoking among non-Western immigrants in France

**DOI:** 10.1093/eurpub/cky230

**Published:** 2018-11-05

**Authors:** M Khlat, S Legleye, D Bricard

**Affiliations:** 1 Mortality, Health, Epidemiology Unit, Institut National d’Études Démographiques (INED), Paris, France; 2 Institut National de la Statistique et des Etudes Economiques (Insee), Montrouge, France; 3 CESP, Faculté de Médecine, Université Paris Sud; Faculté de Médecine UVSQ; Inserm; Université Paris Saclay, Villejuif, France; 4 Institut de Recherche et Documentation en Economie de la Santé (Irdes), Paris, France

## Abstract

**Background:**

Migrants make up a growing share of European populations, and very little is known about the impact of migration on their smoking patterns. We develop a longitudinal analysis of smoking prevalence among native-born and immigrants in France based on retrospective data collected in the 2010 national *Baromètre santé* health survey.

**Methods:**

Analyses concerned 19 578 individuals aged 18–70 years and born in metropolitan France, in the Maghreb or in sub-Saharan Africa. Person-years with and without smoking were reconstructed using migration and smoking histories and analyzed with discrete-time regression models.

**Results:**

Prior to migration, immigrants from both the Maghreb and sub-Saharan Africa had lower smoking prevalence than the native-born of similar birth cohort, age and education. After migration, the prevalence increased over time among Maghrebin men up to levels beyond those of the native-born (odds ratio: 1.54 [1.09–2.17] for 10 years of residence or more), while it remained much lower throughout among men from sub-Saharan Africa (odds ratio: 0.36 [0.19–0.68] for 10 years of residence or more). Starting at extremely low levels, the prevalence in both groups of women rose considerably after migration. Women from sub-Saharan Africa nearly caught up to the native-born (odds ratio: 0.70 [0.37–1.32] for 10 years of residence or more), but this was not the case for those from the Maghreb (odds ratio: 0.52 [0.33–0.81] for 10 years of residence or more).

**Conclusion:**

The findings uncover the low pre-migration prevalence and the diversity of post-migration trajectories. Tobacco control programs targeting recently arrived migrants would contribute to prevent unhealthy assimilation.

## Introduction

In the United States, low smoking-related mortality has been put forward as a significant factor in the migrants’ mortality advantage.^1–4^ Assessing changes in smoking subsequent to migration is therefore a promising avenue for research, both in terms of providing a tangible explanation for the healthy migrant effect and in terms of providing a time frame for attempting to mitigate the deterioration in health that many immigrant groups are experiencing.

Different approaches may be used to investigate lifelong patterns of health in migrants. Research on the erosion of the healthy migrant effect generally builds on cross-sectional analysis, which is questionable given that it ‘potentially confounds duration effects with temporal changes in cohort profiles’.^5^ In certain studies, controlling for cohort of arrival has led to the disappearance of the apparent deterioration in health for some groups.^6^ A longitudinal approach would be more appropriate for integrating in the analysis the health trajectory of the reference native-born population, and the ideal option would be to have pre- and post-migration survey data. As data on health behaviors before and after migration are extremely difficult to gather,^7^ the second-best option would be to build on retrospective data. Smoking is particularly suited to this approach, given the availability of smoking history in certain health surveys.

In a recent review of the literature on smoking among immigrants, a majority of the studies retrieved were from the USA. Studies from Europe were scarce and mostly concentrated in Germany^8–10^ and the Netherlands,^9^^,^^11^^,^^12^ with little recent research in France.^13^^,^^14^ And yet, the share of migrants is growing in European populations, particularly since the 2015 migrant crisis. In particular, France offers excellent potential for exploring smoking disparities by geographical origin, given the diversity and importance of the groups present in the country. In 2014, the share of the immigrant population in France was 8.9% (5.9 million out of 65.8 million).^15^

Recently, we analyzed the smoking prevalence of migrants in the 2010 French national *Baromètre santé* health survey.^16^ Men and women from sub-Saharan Africa and women from the Maghreb (Algeria, Morocco, Tunisia) were found to have a substantially lower smoking prevalence than the native-born, whereas men from the Maghreb had a relatively higher prevalence. We pursue the analysis in the present study by building on the retrospective information to shed light on the smoking transitions that led to the disparities observed in the survey sample. For this purpose, we reconstruct the prevalence of daily smoking among immigrants from North and sub-Saharan Africa at different stages of their life defined in relation with time of migration and compare it to that of the native-born, adjusting for age, birth cohort and education.

This study is one of the first to investigate smoking behavior before and after migration using individual-level data, and the first of this type concerning immigrants in a European country. The findings are interpreted in the light of different theoretical frameworks: the *health behaviour and acculturation hypotheses,*^17^^,^^18^ the cigarette epidemic^19^^,^^20^(and smoking transition models,^11^^,^^21^ and the *healthy/unhealthy assimilation* framework.^5^^,^^22–25^

## Methods

### Data

The 2010 ‘Health Barometer’ is a French nationwide survey that used a two-stage random sampling frame (household/individual) to measure health perceptions and behaviour of the general population.^26^ This investigation was approved by the French Commission on Individual Data Protection and Public Liberties, and all data collected were anonymous and self-reported, with a response rate of 61%. The initial sample of respondents comprised 27 653 individuals aged 15–85 years who answered questions about their migration and smoking histories. Our analyses concerned the subsample of 19 578 individuals born either in metropolitan France or in any of the geographical regions of interest and aged at least 18 years and at most 70 years, the latter cutpoint being used to minimize recall problems and selection biases resulting from differential mortality.^27^

### Measures

The study outcome was daily tobacco smoking, and information on age at starting and cessation were used to construct an accurate age-specific tobacco-use history for each respondent.^28^ To describe life course patterns, we created person-age data with a dichotomous measure of smoking.

Three birth cohorts were defined based on age in 2010: born 1940–55; born 1956–70 and born 1971–92. To make the comparisons of smoking meaningful across cohorts, the ages of retrospective follow-up for the older cohorts are restricted to a maximum of age 39, the upper bound of the youngest cohort, considering initiation after those ages as right-censored. The data were therefore pooled by year of age for each individual from ages 10–39 or current age if lower than 39 years.

Immigrants were categorized based on place of birth and nationality at birth and mother’s and father’s place of birth and nationality. The analysis was limited to the following groups: reference group (born French in metropolitan France from parents born in metropolitan France); born in the Maghreb (Algeria, Morocco or Tunisia) without the French nationality at birth,^29^ referred to hereinafter as the Maghrebins, and; born in sub-Saharan Africa. Reconstructed person-years for migrants were subdivided into the following life stages (time-dependent variable): before migration, 0–5 years after migration, 6–9 years after migration and 10 years or more after migration.

As education is a major determinant of health behaviour, and especially of smoking,^30^ we considered it as a control variable that reflects health advantages emerging early in the life course. Four levels were defined using ISCED (International Standard Classification of Education) standards.^31^ We quantified the education gradient in smoking using the relative index of inequality (RII), a measure that allows comparisons of groups with different education levels.^32^^,^^33^ The RII was computed using ridit scoring for each cohort and gender.^34^ The ridit assigns to each individual the proportion of the overall population that has a higher education plus half of the proportion of the individuals having the same education level, and is therefore a measure of relative education ranging between 0 and 1, from the highest educated to the lowest educated.

### Statistical analyses

A first descriptive analysis compared smoking prevalence at survey time across migrant groups for men and women separately. The models controlled for age at interview, birth cohort and relative educational level.^16^ Longitudinal analyses were conducted using discrete-time regression models. Adjustment of standard errors for clustering within individuals were used for men and women separately to compare, across groups, the life course evolution of smoking prevalence for the age-person data.^35^ The models controlled for age at follow-up, age at follow-up squared, relative educational level,^36^ 15-year birth cohort and relative age in birth cohort group, in order to take into account smoking differentials across generations. Two series of models were run: one model that included only migrants (internal analysis), with the age-person data before migration as the reference, and another model that included both migrants and native-born (external analysis), with native-born as the reference category throughout the life course. Linear and quadratic trend tests were used to evaluate the significance of the shift over the life stages in prevalence of smoking in the internal comparisons (first model). All analyses were conducted with SAS 9.4; the sampling design and survey weights were taken into account with the SURVEY procedures.

## Results

### Sample description

Maghrebins and immigrants from sub-Saharan Africa were younger on the whole than the native-born population, and in turn Maghrebins were older than immigrants from sub-Saharan Africa (table 1). Men from sub-Saharan Africa had a higher median age at arrival than Maghrebin men, and the same applied to women. The median duration of stay in France was far greater for Maghrebins than for immigrants from sub-Saharan Africa, reflecting earlier migration to France for the former. Maghrebins and immigrants from sub-Saharan Africa were less educated than the native-born, and of the two groups, immigrants from sub-Saharan Africa were the most educated. Considering daily smoking at time of survey, the two groups differed widely. There was a very clear gender pattern among Maghrebins, with a higher prevalence in men in comparison with the native-born, and an extremely low prevalence in women. Men and women from sub-Saharan Africa had particularly low levels of prevalence, although higher than the prevalence among Maghrebin women.

**Table 1:** Sample composition and smoking patterns at time of survey (ages 18–70 years)

	Unweighted	Weighted						
Group	Absolute number	Median age at survey	Median age at arrival in France	Median duration of stay in France	% Low education^a^	% High education^b^	% of daily smokers [95%CI]	Odds ratios of smoking^c^ OR [95%CI]
Men								
*Native-born (reference)*	8407	43			23.9	13.0	32.5	1.00
							[31.3–33.7]	
Immigrants from the Maghreb	251	40	20	20	41.2	11.8	41.1	1.22
							[33.7–48.4]	[0.98–1.53]
Immigrants from sub-Saharan Africa	147	39	22	15	34.1	18.5	18.7	0.41
							[10.7–26.7]	[0.28–0.61]
Women								
*Native-born (reference)*	10 356	44			28.8	13.5	26.5	1.00
							[25.5–27.6]	
Immigrants from the Maghreb	221	40	18	24	51.6	10.9	13.6	0.29
							[8.5–18.7]	[0.21–0.41]
Immigrants from sub-Saharan Africa	196	34	19	13	41	12.7	18.8	0.42
							[7.9–29.7]	[0.29–0.59]

a Low education: ISCED 0, 1 or 2 (below upper secondary education).

b High education: ISCED 6 or more (at least a bachelor’s degree or equivalent level).

c Weighted logistic regression with adjustment on: age at interview, birth cohort (1940–55; 1956–70; 1971–92) and relative educational level.

### Longitudinal analysis

The descriptive information on reconstructed person-years with daily smoking and person-years without daily smoking is presented in Supplementary table S1. We developed two angles of analysis. The internal analysis investigates the pattern of change within the group of migrants over time, without considering the native-born. The native-born come into play in the second analysis, which investigates progressive convergence after migration. The results may be summarized as follows (table 2 and Supplementary figures S1–S4):

**Table 2:** Longitudinal retrospective analysis: adjusted odds ratio of daily smoking between ages 10 and 39 years for immigrants according to their lifecycle stage relative to time of migration, based on smoking and migration history (individuals aged 18–70 years)

	Men			Women		
	Odds ratio	95% CI	Trend test	Odds ratio	95% CI	Trend test
Immigrants from the Maghreb						
External analysis^a^						
* Native-born (reference)*	*1.00*			*1.00*		
Before migration	0.61	[0.39–0.95]		0.03	[0.01–0.09]	
0–5 years after migration	0.57	[0.40–0.81]		0.07	[0.03–0.16]	
6–9 years after migration	0.96	[0.65–1.42]		0.17	[0.09–0.33]	
10 years + after migration	1.54	[1.09–2.17]		0.52	[0.33–0.81]	
Internal analysis for immigrants^b^						
* Before migration (reference)*	*1.00*		Linear	*1.00*		Linear
0–5 years after migration	0.92	[0.59–1.43]	*P* = 0.467	2.98	[0.75–11.81]	*P* = 0.284
6–9 years after migration	1.39	[0.76–2.54]	Quadratic:	6.39	[1.58–25.85]	Quadratic
10 years + after migration	1.82	[0.96–3.46]	*P* = 0.021	16.73	[3.88–72.04]	*P* = 0.000
Immigrants from sub-Saharan Africa						
External analysis^a^						
* Native-born (reference)*	1.00			1.00		
Before migration	0.34	[0.17–0.70]		0.03	[0.01–0.08]	
0–5 years after migration	0.25	[0.15–0.44]		0.19	[0.10–0.35]	
6–9 years after migration	0.27	[0.14–0.51]		0.30	[0.17–0.55]	
10 years + after migration	0.36	[0.19–0.68]		0.70	[0.37–1.32]	
Internal analysis for immigrants^b^						
* Before migration (reference)*	1.00		Linear	1.00		Linear
0–5 years after migration	0.64	[0.31–1.33]	*P* = 0.187	8.00	[3.08–20.79]	*P* = 0.001
6–9 years after migration	0.66	[0.24–1.81]	Quadratic:	12.90	[4.49–37.06]	Quadratic
10 years + after migration	0.83	[0.27–2.53]	*P* = 0.824	27.25	[8.24–90.08	*P* = 0.000

a Weighted discrete time logistic regression with adjustment on age at follow-up, age at follow-up^2^, relative educational level, birth cohort group (1940–55; 1956–70; 1971–92) and relative age in birth cohort group. The sample comprised the native-born and immigrants from the Maghreb (top half of the table) and; the native-born and immigrants from sub-Saharan Africa (bottom half of the table). Example: before migration, immigrant men from the Maghreb had an OR of daily smoking of 0.61 compared to the native-born.

b The model described in (a) was run on a sample restricted to the immigrants using the period before migration as the reference. Example: within 5 years from arrival in France, the OR of daily smoking in immigrant men from the Maghreb was 0.92 relative to the period before migration.

In Maghrebins, the internal analysis reveals a steady rise in smoking prevalence starting from 5 years of residence, significant for both genders and much steeper for women, who reach as high an odds ratio as 17 after 10 years or more of residence. In the external analysis, the genders have very distinct patterns. Notably, prevalence in men remains significantly lower than that of the native-born within to 5 years from arrival, then reaches about the same level between 5 and 9 years and continues its rise thereafter, becoming significantly higher after 10 years or more. Prevalence in female migrants is almost null before migration and, although on the rise, remains significantly lower than that of the native-born, even at 10 years or more from arrival.

In migrants from sub-Saharan Africa: in the internal analysis, men have no significant trend throughout their life course. In contrast, women experience a considerable increase, as their prevalence is multiplied by 8 within 5 years from arrival and reaches as high an odds ratio as 28 after 10 years or more of residence in France. The starting level of this impressive rise is close to zero, as shown in the external analysis. This second set of models provides evidence for very low and stable levels in immigrant men relative to the native-born, and of a progressive rise in women, whose levels do not significantly differ from those of the native-born after 10 years or more of residence.

In order to check the robustness of our findings, we re-run the analyses on a constrained subsample of prime-age individuals (25–54 years). The results remained essentially the same (Supplementary table S2).

## Discussion

To our knowledge, only three papers in the literature have developed an analysis of smoking trajectories from pre- to post-migration,^24^^,^^37^^,^^38^ and those contributions concerned immigrants in the United States and Australia. Our study is the first of this kind in a country from Europe and the first to analyze smoking changes after migration in immigrants originating from different regions of Africa. Further to that, our parallel internal and external analyses allowed a comprehensive vision of transition over time. As argued very recently, the ‘most appropriate strategy for adjudicating which health process characterizes the experience of immigrants is through analyzing longitudinal data to document whether the health trajectories of immigrants differ from those of the native population over time’.^5^

Several salient findings emerge from the study. First, men and women from both groups, before migration and upon arrival, had lower smoking rates than their native-born counterparts. The two groups of men, however, differ in their initial levels and post-migration behaviour, whereas the two groups of women differ mainly in their post-migration behaviour. The situation of Maghrebin men is particularly striking, as their prevalence at time of survey was relatively higher than that of the native-born (table 1). For those men, the external analysis revealed very specific dynamics, with lower levels prior to migration and a progressive rise thereafter, leading to definitely higher levels than the native-born after at least 10 years of stay in France. In this group, the upward tendency is also visible in the internal analysis, although the figures do not attain significance due to small numbers. Men from sub-Saharan Africa developed a completely different pattern, as they had very low levels prior to migration, notably lower than those of Maghrebin men and they remained at low levels after migration, even after 10 years or more of residence in France. This discrepancy between the two groups of men suggests an enduring influence of cultural factors in the post-migration context.

Contrasting immigrants from the Maghreb and those from sub-Saharan Africa is of particular interest as those regions of the African continent differ in position within the cigarette epidemic framework. In the 2000s, North African countries, including the Maghreb, were indeed positioned in stage II and sub-Saharan countries in stage I,^39^ both falling behind France which has reached the most advanced stage (IV). According to Constantine et al.,^37^ the Lopez framework stage of country of emigration is ‘as good if not a better predictor of post-migration smoking than measures of acculturation’, as it represents the norms and policies into which the migrants have been socialized. In short, those authors hypothesize that emigrating from a stage 1 country may guard against post-migration smoking, while emigrating from a stage 2 country may entail exposure to higher risks of taking up smoking after migration. Our findings for men (but not for women) accord with this expectation, as those from stage 1 sub-Saharan Africa remain protected from smoking, whereas those from stage 2 North Africa (Maghrebins), who have been raised in a social environment more conducive to smoking, converged and even exceed the native-born levels. The latter case may be considered as a typical case of ‘unhealthy assimilation’.^22^

Another explanatory factor could be the experience of discrimination. Perceiving ethnic discrimination is a stressful experience that may lead to the adoption of adverse coping responses, such as unhealthy behavior.^40^ In the United States, Latinos, Blacks and Asians reporting ethnic discriminations were found to have higher smoking rates than those who did not.^41^ Given that both sub-Saharan and Maghrebin men in France are likely to experience discrimination and socioeconomic disadvantage, their smoking profiles and trends suggest differential responses to perceived discrimination. The importance of the historical and migration background with regard to experience of discrimination has been pinpointed by Silberman and Fournier,^42^ who observe a specific vulnerability of populations originally from zones formerly under colonial domination, i.e. of Maghrebins in comparison with sub-Saharan Africans and Southeast Asians.

Migrant women presently constitute more than half of all migrants in France.^43^ The particularly low levels found for both groups of women prior to migration is consistent with the modification of the cigarette epidemic model proposed by Thun et al.^20^ to allow for gender-specific smoking dynamics. The unfolding of a very sharp ‘unhealthy assimilation’ type of process is all the more impressive as those women had extremely low smoking levels prior to migration. This particular transition is consistent with the expectation of the operant model of acculturation^44^ that ‘behaviours with a low prevalence among initially low-acculturated immigrants increase with acculturation’. Comparing genders, we do not find however that, as sustained by Leung,^24^ ‘immigrant men tend to experience healthy assimilation, whereas immigrant women tend to experience unhealthy assimilation’, as this prediction was grounded in the observation of higher initial smoking among less acculturated male migrants in the USA.

In our study, the gender gap in smoking is much larger for Maghrebins than for immigrants from sub-Saharan Africa, throughout their life course. Given that the smoking prevalence ratio is strongly correlated worldwide with the gender empowerment measure,^45^ the magnitude of this gap supports the idea of the persistence of traditional gender norms long after immigration among Maghrebins, with social disapproval of women’s smoking, as seen in the prevalences of these countries (www.tobaccoatlas.org/topic/prevalence). In our first study based on the same sample,^16^ we found a strong inverse educational gradient in smoking among sub-Saharan females at time of survey, as opposed to no gradient among women from the Maghreb. This is indicative of a less advanced positioning in the cigarette epidemic model for the latter, and may raise concerns about future increases as the epidemic progresses in this group.

The major limitation in our study is the use of retrospective data, and the inherent self-reporting bias, which is likely to differ across groups. There may be differential recall and/or report of smoking histories for migrants and native-born. For example, Maghrebin women may be less at ease reporting smoking. On the other hand, reconstruction of historical rates of smoking prevalence from data on age of smoking initiation and cessation in health surveys has been validated,^28^ and differential mortality was shown to have only small effects on cohort comparisons of educational disparities.^46^

## Conclusion

Migrant women are very much protected from smoking before migration and progressively lose their relative advantage over time, and Maghrebin men undergo the same negative transition. At the opposite, men from sub-Saharan Africa remain at low levels. Further research is needed to better understand the resilience of certain groups, possibly through in-depth qualitative approaches. Within prevention programs targeting recently arrived migrants, special attention should be given both to gender and to the influence of migrants’ backgrounds on the response to anti-smoking messages.

## Funding

This project received funding and administrative support from the Institut national d’études démographiques (INED). Damien Bricard also benefited from a post-doctoral fellowship from INED. 

*Conflicts of interest*: None declared.

**Key points**

- A longitudinal analysis of smoking in immigrants from the Maghreb and from sub-Saharan Africa was developed based on retrospective data from a 2010 national health survey in France.
- Both groups had prior to migration a much lower smoking prevalence than the group of comparable individuals born in France.
- After migration, the prevalence increased in men from the Maghreb but remained low in men from sub-Saharan Africa.
- Prevalence in women from both groups rose sharply and tended to converge with that of women born in France.
- Smoking prevention programs targeting recently arrived migrants are essential for the maintenance of healthy behaviour.

## Supplementary Material

cky230_Supp
