# Supplementary material for: Migration-related changes in smoking among non-Western immigrants in France
Source: Eur J Public Health. 2018 Nov 5;29(3):453–7. doi: 10.1093/eurpub/cky230 (PMC6532831; doi:10.1093/eurpub/cky230)
Supplement: cky230_Supp [file cky230_supp.zip › cky230-Suppl_data/cky230_Supplementary_Figures.docx]

Supplementary Figure 1. Longitudinal retrospective analysis: adjusted odds ratio of daily smoking in immigrants before migration and after migration over time. Men, external analysis (immigrants + native-born)

Supplementary Figure 2. Longitudinal retrospective analysis: adjusted odds ratio of daily smoking in immigrants before migration and after migration over time. Men, internal analysis (immigrants only)

Supplementary Figure 3. Longitudinal retrospective analysis: adjusted odds ratio of daily smoking in immigrants before migration and after migration over time. Women, external analysis (immigrants + native-born)

Supplementary Figure 4. Longitudinal retrospective analysis: adjusted odds ratio of daily smoking in immigrants before migration and after migration over time. Women, internal analysis (immigrants only)
