# Supplementary material for: Migration-related changes in smoking among non-Western immigrants in France
Source: Eur J Public Health. 2018 Nov 5;29(3):453–7. doi: 10.1093/eurpub/cky230 (PMC6532831; doi:10.1093/eurpub/cky230)
Supplement: cky230_Supp [file cky230_supp.zip › cky230-Suppl_data/cky230_Supplementary_Tables.docx]

SUPPLEMENTARY Table 1. *Longitudinal retrospective analysis*: person-years data and prevalence of daily smoking between ages 10 and 39 years in natives and in immigrants according to their lifecycle stage relative to time of migration (individuals aged 18-70 years).

|  | Men | | | Women | | |
| --- | --- | --- | --- | --- | --- | --- |
|  | Unweighted | Weighted | | Unweighted | Weighted | |
|  | Total person-years  between ages 10 and 39 years | Prevalence of daily smoking based on person-years with and without smoking | 95% CI | Total person-years between ages 10 and 39 years | Prevalence of daily smoking based on person-years with and without smoking | 95% CI |
| **Native-born** | 284376 | 35.4 | 35.2-35.6 | 363499 | 24.2 | 24.1-24.4 |
| **Immigrants from the Maghreb** |  |  |  |  |  |  |
| Before migration | 2344 | 22.0 | [19.9-24.1] | 1576 | 0.9 | [0.6-1.2] |
| 0–5 years after migration | 1174 | 34.4 | [31.2-37.7] | 828 | 4.3 | [2.9-5.8] |
| 6–9 years after migration | 740 | 45.6 | [40.2-48.9] | 599 | 8.6 | [6.1-11.0] |
| 10 years + after migration | 3483 | 54.6 | [52.7-56.6] | 3715 | 21.6 | [20.0-23.1] |
| **Immigrants from sub-Saharan Africa** |  |  |  |  |  |  |
| Before migration | 1623 | 15.5 | [13.1-18.0] | 1852 | 1.1 | [0.7-1.5] |
| 0–5 years after migration | 687 | 21.7 | [17.7-25.8] | 925 | 10.1 | [7.8-12.4] |
| 6–9 years after migration | 396 | 25.0 | [19.5-30.4] | 534 | 15.3 | [11.7-19.0] |
| 10 years + after migration | 1472 | 15.0 | [13.0-17.0] | 2044 | 28.4 | [25.1-31.6] |

SUPPLEMENTARY Table 2. *Longitudinal retrospective analysis*: adjusted odds ratio of daily smoking between ages 10 and 39 years for immigrants according to their lifecycle stage relative to time of migration, based on smoking and migration history (individuals aged 25-54 years)

|  | Men | | Women | |
| --- | --- | --- | --- | --- |
|  | Odds ratio | 95% CI | Odds ratio | 95% CI |
| **Immigrants from the Maghreb** |  |  |  |  |
| **External analysis (a)** |  |  |  |  |
| *Native-born (reference)* | *1.00* |  | *1.00* |  |
| Before migration | 0.36 | [0.22-0.60] | 0.01 | [0.00-0.04] |
| 0–5 years after migration | 0.51 | [0.33-0.77] | 0.07 | [0.03-0.15] |
| 6–9 years after migration | 1.08 | [0.69-1.69] | 0.16 | [0.08-0.33] |
| 10 years + after migration | 1.49 | [1.00-2.24] | 0.49 | [0.29-0.82] |
| **Internal analysis for migrants (b)** |  |  |  |  |
| *Before migration (reference)* | *1.00* |  | *1.00* |  |
| 0–5 years after migration | 1.28 | [0.82-2.01] | 6.53 | [2.02-21.10] |
| 6–9 years after migration | 2.54 | [1.25-5.13] | 14.51 | [4.00-52.64] |
| 10 years + after migration | 2.98 | [1.47-6.02] | 37.11 | [8.82-156.08] |
|  |  |  |  |  |
| **Immigrants from sub-Saharan Africa** |  |  |  |  |
| **External analysis (a)** |  |  |  |  |
| *Native-born (reference)* | 1.00 |  | 1.00 |  |
| Before migration | 0.32 | [0.14-0.75] | 0.02 | [0.01-0.06] |
| 0–5 years after migration | 0.36 | [0.20-0.65] | 0.15 | [0.07-0.31] |
| 6–9 years after migration | 0.38 | [0.20-0.70] | 0.27 | [0.13-0.53] |
| 10 years + after migration | 0.51 | [0.26-1.02] | 0.50 | [0.27-0.94] |
| **Internal analysis for migrants (b)** |  |  |  |  |
| *Before migration*  *(reference)* | 1.00 |  | 1.00 |  |
| 0–5 years after migration | 1.09 | [0.50-2.38] | 9.63 | [2.83-32.72] |
| 6–9 years after migration | 1.15 | [0.38-3.50] | 16.13 | [4.21-61.90] |
| 10 years + after migration | 1.58 | [0.46-5.42] | 25.50 | [5.52-117.81] |

(a): weighted discrete time logistic regression with adjustment on age at follow-up, age at follow-up², relative educational level, birth cohort group (1940–1955; 1956–1970; 1971–1992) and relative age in birth cohort group. The sample comprised the native-born and immigrants from the Maghreb (top half of the table) and; the native-born and immigrants from sub-Saharan Africa (bottom half of the table). Example: before migration, immigrant men from the Maghreb had an OR of daily smoking of 0.36 compared to the native-born.

(b): the model described in (a) was run on a sample restricted to the immigrants using the period before migration as the reference. Example: within 5 years from arrival in France, the OR of daily smoking in immigrant men from the Maghreb was 1.28 relative to the period before migration.
